# Two-phase linear relationship of Vitamin D and Vitamin A among children aged 0–14 years: a cross-sectional study

**DOI:** 10.3389/fnut.2025.1539590

**Published:** 2025-04-01

**Authors:** Wenyuan Liu, Qiao Wang, Bibo Mao, Fei Xu, Yefang Ke, Shengying Zhang, Chenbo Zhou, Chunyan Liu, Wenbo Lu, Jishan Zheng

**Affiliations:** ^1^Laboratory of Medicine, The Affiliated Women and Children’s Hospital of Ningbo University, Ningbo, China; ^2^Laboratory of Medicine, Qiu’ga Central Health Clinic, Ningbo, China; ^3^Department of Pediatrics, The Affiliated Women and Children’s Hospital of Ningbo University, Ningbo, China

**Keywords:** Vitamin D, Vitamin A, children, micronutrients, dose–response relationship, public health intervention

## Abstract

**Background:**

Vitamin D and Vitamin A are crucial for children’s immune function, bone health, and cellular growth, but their interrelationship and the impact of various factors remain poorly understood.

**Objective:**

To explore the relationship between Vitamin D and Vitamin A levels in children and identify any critical thresholds.

**Methods:**

A cross-sectional study was conducted from 2018 to 2021 in Ningbo, Zhejiang, China. Participants were children aged 0 to 14 years attending community health service centers for routine health check-ups. Multivariate linear regression analysis was conducted to ascertain the association between serum Vitamin D and Vitamin A levels. A smooth curve fitting approach was employed to analyze the dose–response relationship between Vitamin D and Vitamin A.

**Results:**

A total of 4,752 participants were included. The study revealed significant variations in baseline Vitamin D and Vitamin A levels across different deficiency categories. Mean Vitamin D levels were lowest in the severe deficiency group (4.9 ng/mL) and highest in the normal group (34.8 ± 9.8 ng/mL), while mean Vitamin A levels were lowest in the deficiency group (226.6 ± 72.2 ng/mL) and highest in the normal group (263.3 ± 74.0 ng/mL). The study revealed a two-phase linear relationship with a significant threshold effect at a Vitamin D level of 28.289 ng/mL. Below this threshold, the association between Vitamin D and Vitamin A was strong (*β* = 2.935, 95% CI: 2.173, 3.696), while above the threshold, the association was significantly weaker (*β* = 0.737, 95% CI: 0.413, 1.061). The likelihood ratio test confirmed the significance of this threshold effect (*p* < 0.001).

**Conclusion:**

The study concludes that a significant threshold at 28.289 ng/mL Vitamin D marks a point beyond which the association with Vitamin A levels plateaus, highlighting the importance of this threshold for optimizing vitamin status in children.

## Introduction

Vitamin D and Vitamin A are essential micronutrients that play crucial roles in various physiological processes, including immune function, bone health, cellular growth and differentiation. Deficiencies in these vitamins have been associated with a range of health issues, particularly in pediatric populations where growth and development are most critical. Despite their importance, the relationship between Vitamin D and Vitamin A levels in children remains not fully understood, and the influence of demographic, environmental, and physiological factors on these micronutrients is complex (1, 2).

Vitamin D, often referred to as the “sunshine vitamin,” is synthesized in the skin upon exposure to ultraviolet B (UVB) radiation and is also obtained through diet and supplements. It is well-established that Vitamin D is essential for calcium homeostasis and bone health, with deficiency linked to rickets in children and osteomalacia in adults (3). Recent studies have also highlighted the role of Vitamin D in immune modulation and the prevention of autoimmune diseases, cancers, and cardiovascular diseases (4).

Vitamin A, on the other hand, is a fat-soluble vitamin that is vital for vision, the immune system, and reproductive health. Deficiency in Vitamin A can lead to night blindness, impaired immunity, and increased risk of infections, particularly in children (5). The metabolism of Vitamin A is closely intertwined with that of Vitamin D, suggesting a potential interplay between these two micronutrients that may influence their status in the body (6).

Globally, micronutrient deficiencies are prevalent, particularly in low- and middle-income countries where access to fortified foods and supplements may be limited. Understanding the relationship between Vitamin D and Vitamin A is particularly important given the potential for targeted interventions to improve child health outcomes. Previous studies have examined the individual roles of Vitamin D and Vitamin A, but there is a paucity of research exploring their combined effects and the potential threshold effects in children (7–10). Identifying such a threshold could have significant implications for the development of public health strategies aimed at optimizing Vitamin D and Vitamin A status in children.

This cross-sectional study aims to investigate the relationship between Vitamin D and Vitamin A levels among children aged 0 to 14 years, a population that is particularly vulnerable to the effects of micronutrient deficiencies. We hypothesize that there is two-phase linear relationship between Vitamin D and Vitamin A, with a critical threshold for Vitamin D beyond which the association with Vitamin A levels plateaus. Identifying such a threshold could have significant implications for the development of public health strategies aimed at optimizing Vitamin D and Vitamin A status in children.

By examining the prevalence of Vitamin D and Vitamin A deficiencies and their relationship in a representative sample of children, this study seeks to contribute to the existing body of knowledge and provide insights into the complex interplay between these two vital micronutrients. Our findings may inform clinical practices and public health initiatives aimed at reducing the burden of micronutrient deficiencies in pediatric populations.

## Methods

### Study design

This cross-sectional study aimed to investigate the relationship between Vitamin D and Vitamin A levels among children aged 0 to 14 years. Our sample size was calculated to provide adequate statistical power to identify significant associations, ensuring the robustness of our findings. The recruitment period spanned from the 1st of January 2018, to the 31st of December 2021, allowing us to capture a comprehensive snapshot of the participants’ health status at a specific point in time. This approach enabled us to analyze the prevalence of Vitamin D and their relationship with Vitamin A levels accurately. The study was approved by the Ethical Committee of the Affiliated Women and Children’s Hospital of Ningbo University (No: 20131220), and all participants, as well as their parents or legal guardians, provided signed, informed consent. Children identified with risk Vitamin D or Vitamin A levels will receive further medical evaluation and necessary interventions.

### Study population

Participants were recruited from children aged 0 to14 years who regularly attend community health service centers for routine health check-ups in Ningbo, Zhejiang, China. Inclusion criteria were designed to focus on children who were part of the regular health check-up program at the community health service centers, as this group is particularly relevant for understanding the prevalence and impact of Vitamin A and D status in the pediatric population. Children were included if they were present at the health service centers on the day of the examination and did not have any known chronic diseases or conditions that could influence blood lead levels or Vitamin D status. Exclusion criteria were applied to ensure the study’s findings were not confounded by other health factors. Children with a history of exposure to known environmental contaminants were excluded from the study to isolate the effects of Vitamin A and D on health outcomes. Additionally, children on any medication known to affect bone metabolism were not included, as these medications could potentially skew the results. The same applied to children with any condition that could interfere with the absorption or metabolism of Vitamin D. By recruiting participants from the pool of children who regularly attend community health service centers, the study aimed to capture a representative sample of the pediatric population in terms of Vitamin A and D status. This approach allowed for a more accurate examination of the relationship between these micronutrients and health outcomes in a real-world setting, providing valuable insights for public health initiatives and clinical practices.

### Measurement of Vitamin D and Vitamin A

Blood samples were collected during the physical examination, subsequently stored at −20°C, and then shipped to a centralized laboratory for comprehensive analysis. Serum concentrations of Vitamin D and Vitamin A were ascertained utilizing Inductively Coupled Plasma Mass Spectrometry (ICP-MS) with the AB SCIEX Triple Quad 4500MD Analyzer (Sciex, a division of Danaher Corporation, Framingham, Massachusetts, United States). To maintain the highest standards of accuracy, daily quality control measures were rigorously implemented, employing control materials provided by Hehe (Hefei Hehe Medical Technology Co., Ltd., Anhui, China) for the precise determination of Vitamin D and Vitamin A levels.

Each vitamin’s result is assessed using age-specific reference intervals. Vitamin A levels are classified as “Deficiency” if they fall below the lower limit of the reference interval, “Normal” if they are within the interval, and “Excess” if they exceed the upper limit. Vitamin A levels are categorized as follows:

- 0–6 years: 113.00–647.00 ng/mL
- 7–12 years: 128.00–812.00 ng/mL
- 13–17 years: 144.00–977.00 ng/mL
- ≥18 years: 325.00–780.00 ng/mL

For children under 14 years of age, the reference intervals of Vitamin D are further divided into categories based on the severity of deficiency:

- ≤5.00 ng/mL: Severe deficiency
- 5.01–15.00 ng/mL: Deficiency
- 15.01–20.00 ng/mL: Insufficient
- 20.01–100.00 ng/mL: Normal
- ≥100.01 ng/mL: Excess

These intervals were established through a comprehensive assessment of serum vitamin levels in a diverse group of healthy individuals, ensuring that they are representative of the general population. The laboratory’s quality control measures, including the use of control materials and regular calibration, ensure the accuracy and reliability of the reference intervals.

### Statistical analysis

Data for continuous variables were described as mean ± standard deviation (SD), and data for categorical variables were described as frequencies or percentages. Differences among groups were analyzed by Student *t* test or one-way ANOVA. Qualitative data were analyzed using Chi-square (*χ^2^*) test or Fisher’s exact test as appropriate. To explore the relationship between Vitamin D and Vitamin A levels, smooth curve fitting was adopted, and multivariate linear regression analyses were performed, using unadjusted and multivariate adjusted models to determine the stability of the relationship. Results were adjusted for age and gender in Model 2; age, gender, and season in Model 3. The results are expressed as beta coefficients (*β*) and 95% confidence intervals (CIs). Threshold effect analyses were conducted using two-piecewise regression models to assess the dose–response association of Vitamin D levels and Vitamin A levels, with a likelihood ratio test confirming the significance of the threshold effect.

All the statistical analyses were performed with the statistical software packages R (http://www.R-project.org, The R Foundation) and Free Statistics software versions 1.9.2(Beijing Free Clinical Medical Technology Co., Ltd.). *p* < 0.05 (two-tailed) was declared statistically significant.

## Results

### Baseline characteristics

The baseline characteristics of the study participants stratified by Vitamin D status categories are summarized in Table 1. Out of a total of 4,752 participants, 1 individual was classified with severe deficiency, 176 with deficiency, 374 with insufficiency, and 4,201 had normal Vitamin D levels. Due to the small sample size of the severe deficiency group, it was merged with the deficiency group for the analysis.

**Table 1:** Descriptive characteristics of participants by Vitamin D status categories.

Variables	Total (*n* = 4,752)	Severe deficiency or deficiency (*n* = 177)	Insufficiency (*n* = 374)	Normal (*n* = 4,201)	*P*
Gender, *n* (%)					0.718^b^
Male	2,490 (52.4)	88 (49.7)	193 (51.6)	2,209 (52.6)	
Female	2,262 (47.6)	89 (50.3)	181 (48.4)	1992 (47.4)	
Age, years, Mean ± SD	1.5 ± 1.4	2.8 ± 2.1	2.4 ± 1.8	1.3 ± 1.2	<0.001^a^
Season, *n* (%)					<0.001^c^
Spring	1,324 (27.9)	78 (44.1)	144 (38.5)	1,102 (26.2)	
Summer	1,559 (32.8)	69 (39.0)	132 (35.3)	1,358 (32.3)	
Autumn	1,124 (23.7)	15 (8.5)	44 (11.8)	1,065 (25.4)	
Winter	745 (15.7)	15 (8.5)	54 (14.4)	676 (16.1)	
Vitamin D, ng/mL, Mean ± SD	32.6 ± 11.0	12.3 ± 2.3	17.8 ± 1.3	34.8 ± 9.8	<0.001^a^
Vitamin A, ng/mL Mean ± SD	259.7 ± 74.1	226.8 ± 72.0	235.7 ± 67.5	263.3 ± 74.0	<0.001^a^

a Refers to the one-way ANOVA test.

b Refers to the Chi-square test.

c Refers to the Fisher’s exact test.

There was no significant difference in gender distribution across the Vitamin D status categories (*p* = 0.718). The mean age of participants varied significantly by Vitamin D status categories, with the highest mean age observed in the combined Severe Deficiency and Deficiency group (2.8 ± 2.1 years) and the lowest in the Normal group (1.3 ± 1.2 years; *p* < 0.001). Seasonal variations were also significant, with the highest proportion of participants in the summer season (32.8%) and the lowest in the autumn season (23.7%; *p* < 0.001). Mean Vitamin D levels were significantly different across the categories, with the lowest mean level observed in the combined Severe Deficiency and Deficiency group (12.3 ± 2.3 ng/mL) and the highest in the Normal group (34.8 ± 9.8 ng/mL; *p* < 0.001). Similarly, mean Vitamin A levels varied significantly across the categories, with the lowest mean level observed in the combined Severe Deficiency and Deficiency group (226.8 ± 72.0 ng/mL) and the highest in the Normal group (263.3 ± 74.0 ng/mL; *p* < 0.001).

The distribution of participant characteristics across Vitamin A status categories is presented in Table 2. Out of the total 4,752 participants, 40 were classified with deficiency, 4,710 had normal levels, and 2 were considered excess. Due to the small number of participants in the Excess group, we merged the Normal or Excess groups for analysis.

**Table 2:** Descriptive characteristics of participants by Vitamin A status categories.

Variables	Total (*n* = 4,752)	Deficiency (*n* = 40)	Normal or excess (*n* = 4,712)	*P*
Gender, *n* (%)				0.004^b^
Male	2,490 (52.4)	12 (30.0)	2,478 (52.6)	
Female	2,262 (47.6)	28 (70.0)	2,234 (47.4)	
Age, years, Mean ± SD	1.5 ± 1.4	1.1 ± 1.3	1.5 ± 1.4	0.078^a^
Season, *n* (%)				0.006^c^
Spring	1,324 (27.9)	8 (20.0)	1,316 (27.9)	
Summer	1,559 (32.8)	16 (40.0)	1,543 (32.7)	
Autumn	1,124 (23.7)	16 (40.0)	1,108 (23.5)	
Winter	745 (15.7)	0 (0)	745 (15.8)	
Vitamin D, ng/mL, Mean ± SD	32.6 ± 11.0	23.6 ± 12.0	32.7 ± 11.0	<0.001^a^
Vitamin A, ng/mL, Mean ± SD	259.7 ± 74.1	98.4 ± 15.1	261.1 ± 72.9	<0.001^a^

a Refers to the Student *t* test.

b Refers to the Chi-square test.

c Refers to the Fisher’s exact test.

Gender distribution varied significantly across Vitamin A status categories, with a higher proportion of females in the deficiency group compared to males (30% females vs. 70% males in the deficiency group, *p* = 0.004). The mean age of participants was not significantly different across Vitamin A status categories (*p* = 0.078). Seasonal distribution showed significant differences, with a higher proportion of participants in the summer and autumn seasons within the deficiency group (*p* = 0.006). Vitamin D levels were significantly lower in the deficiency group compared to the normal and excess groups (*p* < 0.001). Mean Vitamin A levels were markedly lower in the deficiency group (98.4 ± 15.1 ng/mL) and higher in the normal and excess group (261.1 ± 72.9 ng/mL), indicating a significant difference across the categories (*p* < 0.001).

### Association between Vitamin D and Vitamin A

The results of the regression analysis examining the association between Vitamin D and Vitamin A levels are detailed in Table 3. The analysis was conducted in three models with increasing adjustments for covariates.

**Table 3:** Association between Vitamin D and vitamin A.

Variable	Model 1, *β* (95% CI)	Model 2, *β* (95% CI)	Model 3, *β* (95% CI)
Vitamin D, ng/mL	0.97 (0.78~1.16) <0.001	1.34 (1.15~1.54) <0.001	1.36 (1.16~1.56) <0.001
Quintiles Q1 (<24.95 ng/mL)	0 (Ref)	0 (Ref)	0 (Ref)
Quintiles Q2 (≥24.95, <31.87 ng/mL)	18.83 (12.93~24.72) <0.001	25.83 (19.92~31.73) <0.001	26.19 (20.25~32.14) <0.001
Quintiles Q3 (≥31.87.0, <39.20 ng/mL)	24 (18.11~29.9) <0.001	33.85 (27.85~39.85) <0.001	34.31 (28.21~40.41) <0.001
Quintiles Q4(≥39.20 ng/mL)	29.47 (23.57~35.37) <0.001	40.67 (34.61~46.73) <0.001	41.48 (35.33~47.64) <0.001
*p* for trend	<0.001	<0.001	<0.001
Gender			
Male	0.91 (0.64~1.18) <0.001	1.33 (1.05~1.6) <0.001	1.34 (1.06~1.62) <0.001
Female	1.03 (0.76~1.3) <0.001	1.36 (1.08~1.64) <0.001	1.39 (1.11~1.67) <0.001
Age group			
0~≤6 months	2.67 (−0.59~5.93) 0.123	2.7 (−0.64~6.04) 0.129	2.14 (−1.67~5.94) 0.286
6~≤12 months	1.33 (1.08~1.59) <0.001	1.33 (1.08~1.59) <0.001	1.35 (1.1~1.61) <0.001
1~≤3 years	1.4 (1.02~1.77) <0.001	1.4 (1.03~1.78) <0.001	1.31 (0.92~1.71) <0.001
3~≤6 years	2.02 (1.56~2.49) <0.001	2.03 (1.56~2.5) <0.001	2.12 (1.64~2.6) <0.001
6~≤14 years	4.28 (2.37~6.19) <0.001	4.28 (2.36~6.21) <0.001	4.64 (2.67~6.62) <0.001
Season			
Spring	1.01 (0.68~1.34) <0.001	1.56 (1.22~1.9) <0.001	1.56 (1.22~1.9) <0.001
Summer	0.84 (0.52~1.17) <0.001	1.26 (0.93~1.58) <0.001	1.26 (0.93~1.58) <0.001
Autumn	1.17 (0.74~1.61) <0.001	1.81 (1.39~2.24) <0.001	1.81 (1.39~2.24) <0.001
Winter	0.94 (0.4~1.47) <0.001	1.49 (0.92~2.07) <0.001	1.49 (0.92~2.07) <0.001

Model 1, No covariates were adjusted; Model 2, Gender and age were adjusted; Model 3, Gender, age and season were adjusted.

In Model 1, every 1 ng/mL increase in Vitamin D was associated with a significant increase in Vitamin A by 0.97 units (95% CI: 0.78, 1.16). After adjusting for gender and age in Model 2, the association remained significant with a *β* of 1.34 (95% CI: 1.15, 1.54). Further adjustment for season in Model 3 did not substantially change the estimate, yielding a *β* of 1.36 (95% CI: 1.16, 1.56).

When analyzing Vitamin D levels by quintiles, compared to the reference category (Quintile Q1: <24.95 ng/mL), participants in higher quintiles showed significantly higher values of Vitamin A. Specifically, Quintile Q2 and Q3 had increases of 18.83 (95% CI: 12.93, 24.72) and 24 (95% CI: 18.11, 29.9) respectively in Model 1. The highest quintile, Q4, showed the largest increase of 29.47 (95% CI: 23.57, 35.37). These findings were consistent across all models. The *p*-values for trend across all models were less than 0.001, indicating a consistent and significant linear trend.

Stratified analyses by gender demonstrated significant associations for both males and females, with higher estimates in the adjusted models. Age group stratification revealed significant associations across all age groups, with the largest increases observed in the 6 to ≤14 years age group. Seasonal variation also played a significant role in the association, with the highest *β* estimates observed in the summer and autumn seasons.

**Figure 1:**
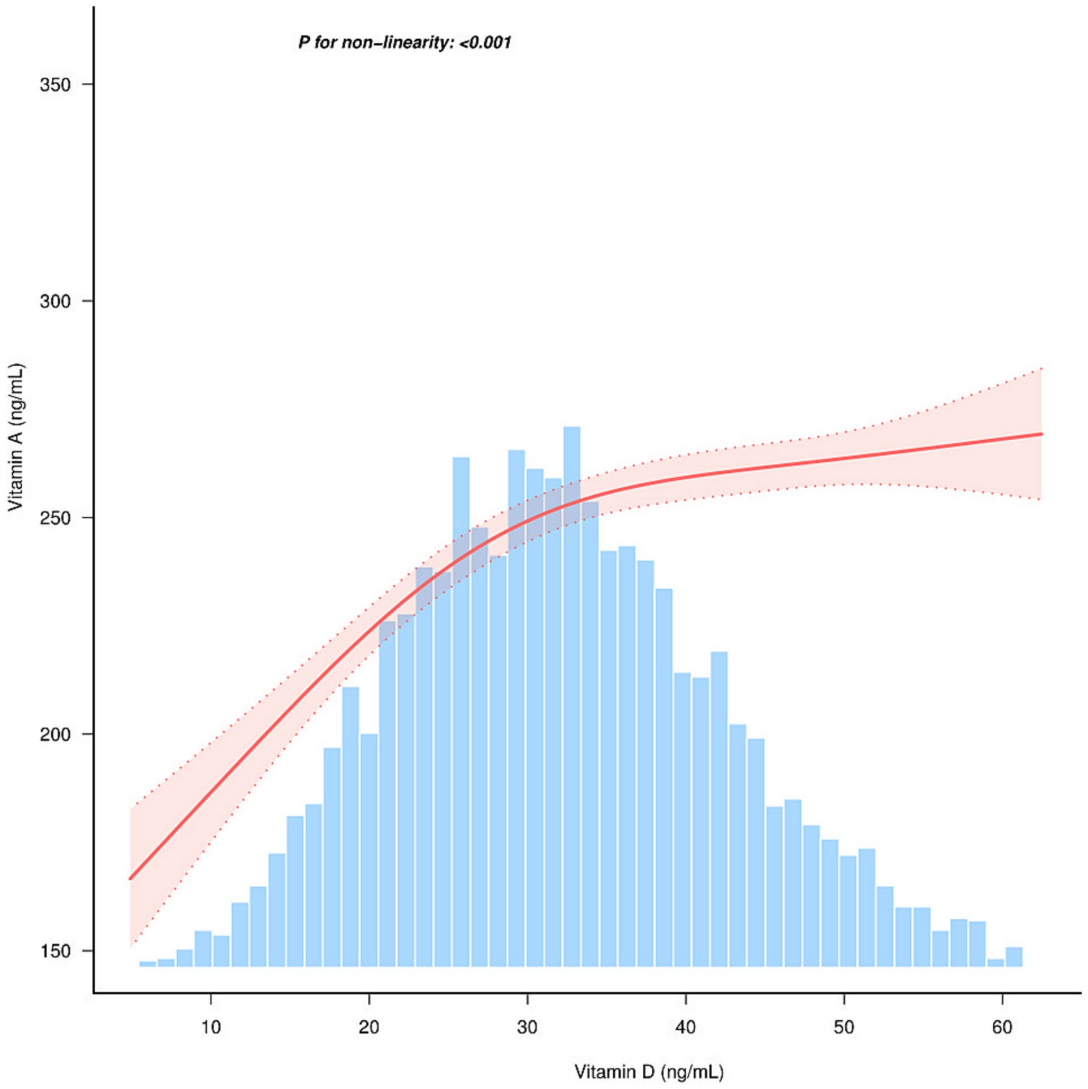
Relationship between Vitamin D and Vitamin A. Solid and dashed lines represent the predicted value and 95% confidence intervals. They were adjusted for gender, age, and season.

Figure 1 and Table 4 present the results of the analysis examining the relationship between Vitamin D and Vitamin A levels, with a focus on the threshold effect.

**Table 4:** Threshold effect analysis of the relationship of Vitamin D and Vitamin A.

Vitamin D	*β* (95% CI)	*P-*value
<28.289 ng/mL	2.935 (2.173, 3.696)	<0.001
≥28.289 ng/mL	0.737 (0.413, 1.061)	<0.001
Likelihood ratio test	-	<0.001

CI, confidence interval. Adjusted for gender, age, and season.

The association between Vitamin D levels and Vitamin A was linear (*p* < 0.001) in the restricted cubic spline model (Figure 1). The two-piecewise regression models have uncovered a notable threshold effect in the relationship between Vitamin D levels and Vitamin A. Table 4 provides the threshold effect analysis of the relationship between Vitamin D and Vitamin A, adjusted for gender, age and season. The analysis reveals a significant threshold effect at a Vitamin D level of 28.289 ng/mL. For participants with Vitamin D levels below the threshold of 28.289 ng/mL, the beta coefficient (*β*) is 2.935 (95% CI: 2.173, 3.696), indicating a strong association with Vitamin A levels. In contrast, for those with Vitamin D levels at or above the threshold, the beta coefficient is significantly lower at 0.737 (95% CI: 0.413, 1.061). This suggests that the relationship between Vitamin D and Vitamin A levels changes at the specified threshold, with a marked decrease in the strength of the association at higher Vitamin D levels. The likelihood ratio test further confirms the significance of this threshold effect (*p* < 0.001), reinforcing the two-phase linear nature of the association between Vitamin D and Vitamin A levels.

## Discussion

Our study provides a comprehensive examination of the intricate relationship between Vitamin D and Vitamin A levels in children aged 0–14 years, uncovering a two-phase linear relationship with a critical threshold for Vitamin D beyond which the association with Vitamin A levels plateaus. This threshold effect is of significant importance for public health strategies aimed at optimizing the status of these vitamins in children. The baseline characteristics of our study participants, as detailed in Table 1, reveal substantial variations in Vitamin D and Vitamin A levels across different deficiency categories. The observed differences in mean levels of these vitamins by gender, age, and season highlight the influence of demographic and environmental factors on micronutrient status.

The higher mean age in the severe deficiency group and seasonal variations suggest a complex interplay between Vitamin D synthesis, sunlight exposure, and nutritional intake, warranting further investigation. Older children may have lower Vitamin D levels due to increased growth rates and skeletal mineralization demands, which can deplete body stores of Vitamin D (11). Additionally, the observed seasonal variations align with Holick’s findings that UVB radiation, which affects cutaneous Vitamin D production, varies with season and latitude (12). Gender differences in Vitamin D levels may be influenced by outdoor activity levels, clothing habits, hormonal variations, and other factors such as skin pigmentation and sun protection behaviors (13). Vitamin D synthesis in the skin is a photosensitive process that depends on balancing UVB exposure and skin protection, as noted by Holick (14). This balance is crucial as overexposure to UVB can cause skin damage, while insufficient exposure leads to inadequate Vitamin D synthesis, highlighting the need for public health strategies that optimize sun exposure while minimizing skin cancer risks.

The association analysis indicates a robust relationship between Vitamin D and Vitamin A levels, even after adjusting for multiple covariates. The consistent increase in the beta coefficients across the three models suggests that higher Vitamin D levels are positively associated with higher Vitamin A levels. This association may reflect a common underlying mechanism or pathway through which both vitamins contribute to similar physiological processes, such as immune function and bone health.

The threshold effect observed in our study, with a significant breakpoint at 28.289 ng/mL of Vitamin D, suggests that the relationship between Vitamin D and Vitamin A is not uniform across all levels of Vitamin D. Below this threshold, the association is markedly stronger, indicating that Vitamin D may play a more critical role in modulating Vitamin A levels when Vitamin D levels are low. This phenomenon can be explained by several biological mechanisms. Vitamin D is crucial for maintaining calcium homeostasis, which directly influences the absorption and metabolism of Vitamin A. Deficiencies in Vitamin D can impair calcium absorption, thereby indirectly affecting Vitamin A status through alterations in the activity of calcium-binding proteins involved in micronutrient transport (15). Vitamin D deficiency can lead to rickets in children and osteomalacia in adults, highlighting its critical role in regulating calcium levels in the body. The synthesis of Vitamin D involves the skin, liver, and kidneys and is regulated by parathyroid hormone (PTH) and blood calcium levels. This complex interplay is essential for maintaining overall calcium homeostasis and, consequently, the proper absorption and metabolism of Vitamin A (16, 17). Vitamin D also has well-documented effects on immune function, modulating the expression of cytokines and other immune mediators that can influence Vitamin A metabolism and retinoid signaling (18). In conditions of infection or inflammation, both Vitamin D and Vitamin A play critical roles, and their interaction may be particularly important when Vitamin D levels are low. Genetic variations in enzymes involved in the metabolism of Vitamin D and Vitamin A can influence their levels and interactions (19). For example, variations in the gene encoding retinol-binding protein 4 (RBP4) have been associated with altered Vitamin A metabolism (20). Similarly, genetic factors influencing the hydroxylation of Vitamin D may affect its levels and subsequent interactions with Vitamin A. Lastly, the synthesis of Vitamin D in the skin is dependent on UVB exposure, and variations in sunlight exposure can significantly affect Vitamin D levels (21). In regions with limited sunlight, Vitamin D deficiency is more prevalent, and the interaction between Vitamin D and Vitamin A may be more pronounced (22). Additionally, environmental factors such as air pollution and lifestyle factors (e.g., time spent outdoors) can influence both Vitamin D synthesis and Vitamin A status (23–25). The observed threshold effect may be influenced by the interplay between environmental factors and the metabolic demands of Vitamin D. For example, in regions with high air pollution or limited sunlight, Vitamin D deficiency is more prevalent, which can also impact Vitamin A status. Therefore, understanding these influences is crucial for developing strategies to optimize Vitamin D levels and, by extension, support Vitamin A status (26).

Our study’s identification of a nonlinear relationship and an inflection point for Vitamin D levels in children has significant clinical implications. This finding underscores the necessity of maintaining Vitamin D within an optimal range to support children’s health, especially for high-risk groups like older children and those with limited sun exposure. Our findings provide a concrete threshold to guide clinical monitoring and interventions, helping clinicians target Vitamin D supplementation and dietary advice to ensure children’s Vitamin D levels are neither too high nor too low. Public health strategies should promote optimal sun exposure for Vitamin D synthesis while minimizing skin cancer risks. Tailored interventions should address the needs of children with low Vitamin D, considering interactions with Vitamin A. The inflection point marks a critical threshold where Vitamin D’s impact on health outcomes plateaus, offering a target for clinical intervention. This knowledge can inform targeted interventions to optimize Vitamin D levels, potentially improving pediatric health outcomes.

Our study provides valuable insights into the relationship between Vitamin D and Vitamin A levels; however, several limitations should be noted. The cross-sectional design means that measurements were taken at a single time point, limiting our ability to infer causality or assess the dynamic changes in these micronutrients over time. Additionally, our study population was limited to children in Ningbo, Zhejiang, which has a specific climate and dietary patterns. This may restrict the generalizability of our findings to other regions with different climates, latitudes, or dietary habits. Finally, while we excluded children with chronic diseases or relevant medications, residual confounding from unmeasured factors such as genetic variations, socioeconomic status, or micronutrient supplementation practices could influence the observed associations. Future research should address these limitations through longitudinal studies and by considering a broader range of potential confounders.

## Conclusion

Our study confirms the significant interaction between Vitamin D and Vitamin A in children and identifies a critical threshold of 28.289 ng/mL for Vitamin D, beyond which the association with Vitamin A levels plateaus. This threshold underscores the importance of optimal Vitamin D levels in modulating Vitamin A status, particularly in high-risk groups such as older children and those with limited sun exposure. Future longitudinal studies are warranted to confirm these findings and investigate whether fluctuations in Vitamin D levels predict Vitamin A deficiencies or other health outcomes over time. Such research could elucidate causal pathways and inform targeted interventions for at-risk populations. Our findings pave the way for these efforts and further mechanistic research into the interplay between these micronutrients.

## Data Availability

The original contributions presented in the study are included in the article/supplementary material, further inquiries can be directed to the corresponding authors.
